# Malaria vaccines: the 60-year journey of hope and final success—lessons learned and future prospects

**DOI:** 10.1186/s41182-023-00516-w

**Published:** 2023-05-17

**Authors:** Amal A. El-Moamly, Mohamed A. El-Sweify

**Affiliations:** 1 Department of Medical Parasitology, Faculty of Medicine, Suez Canal University, Ismailia, Egypt; 2 Department of Medical Microbiology and Immunology, Faculty of Medicine, Suez Canal University, Ismailia, Egypt

**Keywords:** Malaria, *Plasmodium falciparum*, Vaccine, Development, History, Challenges, Approval, RTS,S, Implementation program, Pre-erythrocytic vaccines, Erythrocytic vaccines, Blood-stage vaccines, Transmission blocking vaccines, Control, Elimination

## Abstract

**Background:**

The world has made great strides towards beating malaria, although about half of the world population is still exposed to the risk of contracting malaria. Developing an effective malaria vaccine was a huge challenge for medical science. In 2021 the World Health Organization (WHO) approved the first malaria vaccine, RTS,S/AS01 vaccine (Mosquirix™), for widespread use.

**Main abstract body:**

This review highlights the history of development, and the different approaches and types of malaria vaccines, and the literature to date. It covers the developmental stages of RTS,S/AS01 and recommends steps for its deployment. The review explores other potential vaccine candidates and their status, and suggests options for their further development. It also recommends future roles for vaccines in eradicating malaria. Questions remain on how RTS,S vaccine will work in widespread use and how it can best be utilized to benefit vulnerable communities.

**Conclusion:**

Malaria vaccines have been in development for almost 60 years. The RTS,S/AS01 vaccine has now been approved, but cannot be a stand-alone solution. Development should continue on promising candidates such as R21, PfSPZ and *P. vivax* vaccines. Multi-component vaccines may be a useful addition to other malaria control techniques in achieving eradication of malaria.

## Background

Developing an effective malaria vaccine has been a huge challenge for medical science and the world has made great strides towards beating malaria. It is one of the oldest of mankind’s deadliest enemies and is still a major health problem in many countries. According to the World Health Organization’s 2020 World Malaria Report, there were 229 million cases reported in 2019 and 409,000 deaths [1]. Children younger than 5 years old made up 67% of the deaths, and the disease is still killing 1 child every 2 min. In 2019, about half of the world population was exposed to the risk of contracting malaria [1]. Sub-Saharan Africa suffers the most, accounting for more than 90% of malaria cases and deaths annually.

Recent advances in control efforts have introduced many advances, including highly effective therapies, such as the artemisinin combination therapy, and rapid diagnostic tests. The wider use of insecticide-treated bed nets, various vector control measures, and preventive intermittent chemotherapeutic courses to vulnerable individuals, have all helped reduce the incidence of malaria. However, this reduction has recently slowed and the incidence may be increasing again [1]. According to the UN’s Sustainable Development Goals, the targets of number 3 (to ensure well-being and promote healthy lives for all individuals at all ages) are a 90% reduction in malaria incidence and mortality, and malaria elimination in at least 35 endemic countries by 2030 [2]. This increased concern requires extra tools to fight the disease. The approval of the RTS,S vaccine is just in time to maximize the public health benefit of all these efforts [3].

October 6, 2021 was a historic day, when the WHO approved the first malaria vaccine and parasitic vaccine, RTS,S/AS01 (RTS,S, also known as Mosquirix™) for widespread use. The vaccine significantly reduces total malaria cases, and the deadly form of the disease among young children [4]. Given that a malaria vaccine has been under development since the 1960s, this is considered to be one of medicine's biggest achievements [5]. The new vaccine was developed by GlaxoSmithKline (GSK), a British pharmaceutical company, and was first shown to be effective in 2015. We hope its approval will revived the battle against malaria. The WHO has recommended widespread use of the RTS,S vaccine to immunize children in regions with moderate-to-high transmission of *P. falciparum* malaria, i.e. mainly sub-Saharan Africa [5]. This decision is justified by good results from a pilot program implemented in three African countries (Kenya, Ghana and Malawi). The pilot program started in 2019 and has vaccinated some 800,000 young children [6]. The RTS,S vaccine was widely accepted by the communities involved and now it has been on approved, it will be routinely delivered in national childhood healthcare programs.

According to Dr. Tedros Adhanom Ghebreyesus, WHO Director-General, the long-awaited vaccine for children is a breakthrough for science, child health, malaria control, and a gift to the world. This first-ever vaccine for a parasite is a game changer that brings us one step closer to a malaria-free world. Using this vaccine in addition to existing prevention tools could save tens of thousands of young lives each year and change African lives forever [6].

"It has been a long way of hope for an effective malaria vaccine and now for the first time ever, we have such a vaccine recommended for widespread use…. Today’s recommendation offers a glimmer of hope for Africa, which shoulders the heaviest burden of the disease, and we expect many more African children to be protected from malaria and grow into healthy adults.” Dr. Matshidiso Moeti, WHO Regional Director for Africa [6].

The search for malaria vaccines was started in 1965 by immunologist Dr. Ruth Nussenzweig [7], although many scientists and companies also dedicated their lives to ending malaria [8]. There are now many potential vaccine candidates.

This review highlights the history of malaria vaccines, the different approaches to their development and different types of vaccines. It mainly looks at the stages of RTS,S/AS01 vaccine and recommends steps for deploying RTS,S. It generally explores other potential vaccine candidates, their status and challenges, and suggests prospects for further development. It also makes recommendations on the role of future vaccines in eradicating malaria.

A literature review searched PubMed, Scopus and Clarivate Web of Science up to 30 December 2021 for articles on malaria vaccine development. Terms included were: “malaria”, “WHO", “*Plasmodium falciparum*”, “RTS,S”, “RTS,S/AS01”, “Mosquirix™”, “vaccine”, “vaccination”, " approval", "pilot program", “pre-erythrocytic vaccine”, “erythrocytic vaccine”, "blood stage vaccine", “transmission blocking vaccine”, “circumsporozoite protein”, "whole sporozoite", "sporozoite subunit", "vectored vaccines", "R 21", "PfSPZ", and combinations of these. The initial search and screening of all papers was carried out by a contributor (OME), and the authors (AAE) and (MAE) re-assessed the content of all papers. Subjectively, 131 articles were included based on their relevance to the study objectives and aims. Preference was given to articles that comprehensively and/or appropriately covered the topics of interest. Additional articles were identified by visiting relevant websites, e.g. WHO, PATH global health organization (formerly Program for Appropriate Technology in Health) and major journals. No language restrictions were used.

**Main text**

## Why did it take so long to develop a malaria vaccine?

The development of malaria vaccines has taken almost 60 years of hard work. The journey that started in the early 1960s was inspired by the remarkable success of vaccines against polio, measles, diphtheria, tetanus, rabies and other diseases. The complete eradication of smallpox in humans proved the potential of this approach to reduce the global burden of infectious diseases [9].

Initial attempts to develop a malaria vaccine resulted in great frustration. Researchers realized that vaccines against this disease would be challenging to develop and it became increasingly clear that it is due to a clever parasite. Impediments to successful malaria vaccination are multifactorial. The main difficulties were the malaria parasite’s (*P. falciparum*) extremely complex biology, life cycle and genome in addition to the parasite’s evasion of the human immune system and the absence of sterile immunity to the disease [10].

It is noteworthy that parasites are difficult to develop vaccines against. The recently approved RTS,S malaria vaccine is the only successful vaccine for a parasitic disease so far. Vaccines against parasites are difficult to develop because the human immune response to parasites is unique, due to their complicated life cycle and the immune escape mechanisms expressed by different parasites. Growing a sufficient number of whole parasites to generate an immune response is also a major challenge in order to develop a vaccine, despite the recent success with malaria [11]. To overcome this obstacle, efforts were directed at obtaining many types of parasite antigens (mainly proteins) or from vectors trying to induce a protective immune response [12]. It was also a major challenge to generate an adequate immune response based on small antigens that represented less than 1% of the whole parasite [8].

Factors like the complex life cycle, genetic diversity, pathophysiologic complexity, and the parasite’s various immune escape mechanisms lead to antigenic variations [13]. Because of the high number of polymorphisms or allele-specific variations in the proteins, single protein-based vaccines had limited success [14]. The *Plasmodium* parasites’ genetic make-up consists of about 5400 coding genes, and with the absence of adequate natural human immunity against the disease, these make malaria unique from other microbial pathogens for which successful vaccines have been developed [15]. Moreover, malaria has been mutating for 30 million years, and after a person has contracted malaria, they can only acquire partial immunity—unlike a virus which can elicit solid immunity [5]. The *Plasmodium* genome is much larger and more complex than bacterial or viral genomes. Its complicated life cycle has an asexual phase (schizogony) in humans and a sexual phase (gametogony) in mosquitoes [16]. Antigen expression is phase-specific [10] so different immune system arms are required depending on the parasite’s extracellular or intracellular location and distinct immunogenic properties. The protective antibodies against sporozoites (sexual forms transmitted by the mosquito in man) fail to recognize merozoites (asexual erythrocytic stages that cause clinical malaria). This means that if only one sporozoite evades the antibodies released as response to a vaccine, we can expect approximately 10,000–40,000 merozoites to be active after one week to start clinical disease. This poses a big challenge to developing a highly effective vaccine to malaria [8, 17, 18].

Targeting the erythrocyte stages of the life cycle is also difficult as they are subject to antigenic variation and can easily evade the human immune system [10]. Another challenge to developing a malaria vaccine is the ability of *P. vivax* and *P. ovale* to produce dormant hypnozoite stages in the liver, which are not tackled by the blood-stage vaccine candidates [10]. Another form of the parasite’s effective immune evasion is its capacity to mimic epidermal cell antigens and induce antigenic variations in blood cells and thereby inhibit apoptosis in liver cells [10]. Thus, there is no solid natural immune response in the course of malaria; after years of exposure only a weak and partial immunity can develop. Since natural immunity is directed against a wide-range of erythrocytic antigens, immunological studies have found it difficult to identify the best antigens for developing an ideal vaccine [19]. In addition, the species-specificity of *P. falciparum* and *P. vivax,* which do not infect most small animals or old world macaques that are used for models of vaccine evaluation, also posed a challenge. *Plasmodium* species that infect these animals are different from those that infect humans [10].

Another problem in developing a malaria vaccine was financial. Malaria mainly affects people in countries with limited resources, where there is little motivation or reward for investing in vaccines; instead manufacturers continued targeting industrialized first-world markets [20, 21]. Malaria-endemic countries lack a robust healthcare infrastructure, so they present less attractive investment markets to large corporations, but put their efforts into vaccines for less serious diseases that can make a profit in Western markets [5, 10]. In addition, investing in parasitic vaccines carries a higher financial risk because they are significantly more difficult to develop than virus vaccines. [5]. Malaria vaccine development has therefore suffered from less funding and fewer research initiatives [5]. Apart from the huge investments made by the Bill and Melinda Gates Foundation, only GSK has invested in a malaria vaccine. However, the evolution of public–private partnerships, such as the Malaria Vaccine Initiative of the Bill and Melinda Gates Foundation, offered hope for enhanced malaria research [10].

The strict regulations imposed by national vaccine licensing authorities were another barrier to the development of a vaccine. These increase the cost of clinical development pathways heavily. The pharmaceutical industry therefore has to charge more for a new vaccine to recoup its investment if it is not subsidized by non-government organizations and public–private partnerships [20, 21].

## History of malaria vaccine development

The history of modern malaria vaccine began in the early 1960s with experimental studies on primates, rodents and humans to test irradiated sporozoites [7, 22]. The first promising results were documented by Clyde et al. in the 1970s [23] who found high protective efficacy from using radiation-attenuated sporozoites in persons of a high number of bites by irradiated infectious mosquitoes. Later, complete protection was demonstrated by using attenuated sporozoites using gamma radiation on infected mosquitoes in 2002 [24].

The promised major component of the sporozoite coat (circumsporozoite protein) was identified and its coded gene cloned and sequenced in the 1980s [25]. At that time, a range of blood-stage antigens was also identified and expressed, raising hopes for a blood-stage vaccine. However, preliminary trials did not show promising results for the candidate antigens and their efficacy on sporozoite challenge was statistically insignificant [26]. In 1988, asexual stage vaccine (SPf66 candidate), emerged in Colombia and had an acceptable efficacy in humans and animals (new-world monkeys) [27]. This peptide-based vaccine was interesting, but disappointing when field studies in Africa and Asia demonstrated insufficient efficacy [8]. However, the early studies on SPf66 and on sporozoite-based and mosquito-based vaccines led to further field technologies that were used to assess later vaccines (see section “Types of malaria vaccines”).

### Types of malaria vaccines

**Fig. 1:**
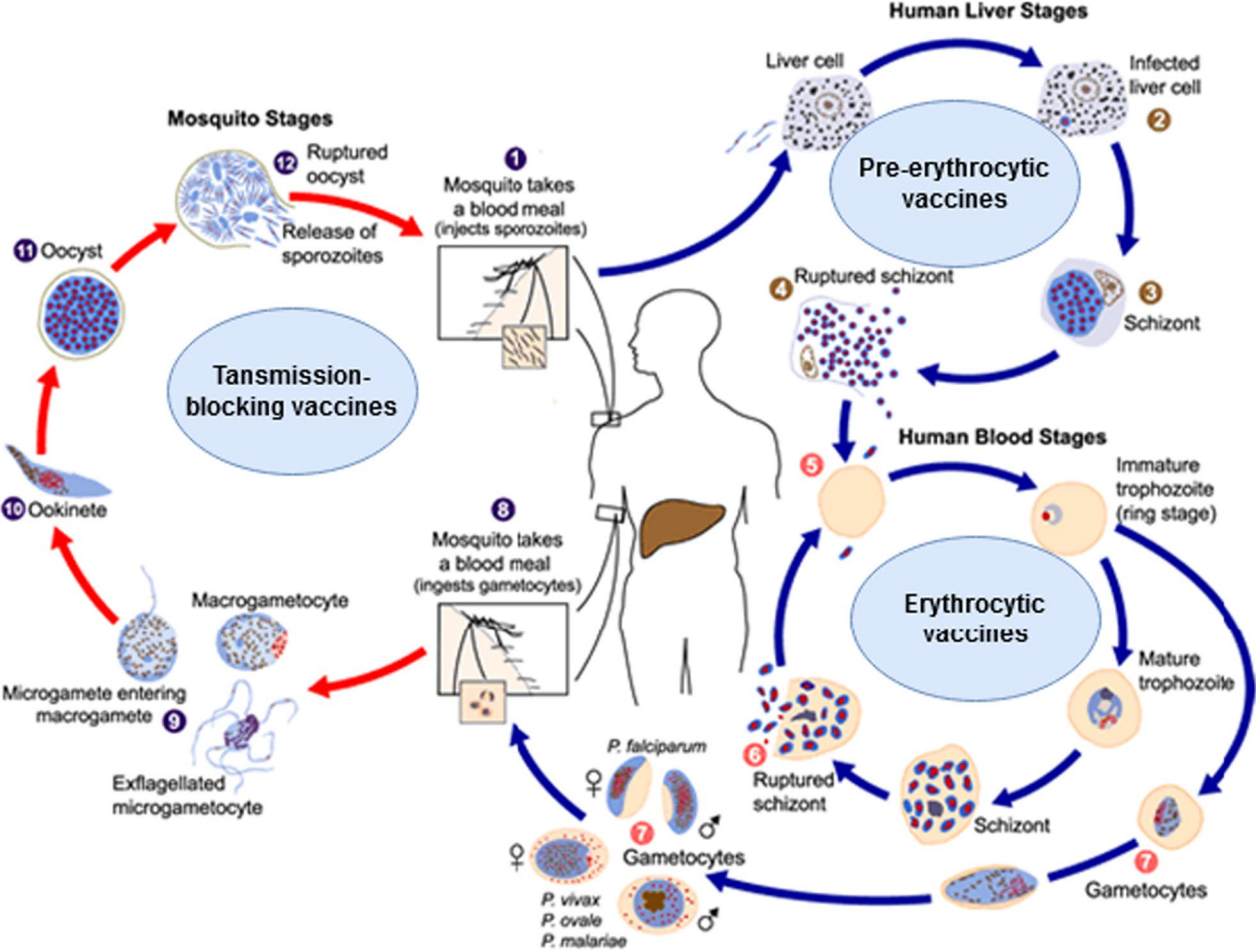
Life cycle of the malaria parasite and the vaccine types targeting various life cycle stages. Image courtesy of DPDx, Centers for Disease Control and Prevention (https://www.cdc.gov/dpdx). Image was adapted to show various malaria vaccines' target stages. Detailed information on malaria's life cycle is available on the provided website

Malaria vaccines are categorized according to the parasite’s targeted developmental stage: pre-erythrocytic vaccines (anti-infection), erythrocytic vaccines, and transmission-blocking vaccines (Fig. 1). Most malaria vaccines target one of these three phases [8, 17, 18, 21], although some target two or three phases. A wide range of new vaccine technologies is now used.

#### Pre-erythrocytic vaccines (PEVs)

Experts believe that the best vaccine is one that attacks the early stages to completely block the development of subsequent stages, infection and transmission [3]. The pre-erythrocytic (liver stage) vaccines target sporozoites, i.e. the sexual forms transmitted by mosquito to man. PEVs are expected to induce antibodies to sporozoite surface antigens (needed to attack sporozoites in the skin and blood vessels) and prevent the invasion of the liver cells, and also induce a T-cell response needed to clear infected liver cells [17]. PEVs attack the critical early phase in which the sporozoites infect a few liver cells and need approximately one week of development in the liver phase—which gives enough time for the vaccine to act. However, the infected hepatocytes, unlike the infected erythrocytes, express parasite antigens that can induce T-cells to target and kill these cells, thus preventing merozoites being released into the blood [8, 17, 21, 28–32]. Thus, PEVs with a high efficacy offer the opportunity to completely eradicate the hepatic pre-erythrocytic stages and prevent further infection [17]. PEVs are thought to be more effective vaccines than those directed against later stages [33]. They contain whole sporozoites or antigenic subunits of the circumsporozoite proteins [8, 34].

##### Whole sporozoite vaccine (WSV)

Whole sporozoites are managed by radiation or by chemical or genetic attenuation, and are then given to recipients by mosquito bites. After entering the liver, they partially develop in the hepatocytes and induce a broad immune response, including CD4- and CD8-T cells, and antibodies, without causing disease [11, 24, 35–37]. Although whole sporozoite vaccines have induced sterilizing immunity to challenge sporozoites in humans, no further steps have been taken to complete the production of this type of vaccine [38]. Attenuating sporozoites by irradiation is costly and not easily applicable in a wider setting [24]. However, there is now renewed interest in the whole-organism vaccine as a result of a highly successful human trial using experimental sporozoite inoculation with chloroquine prophylaxis [39, 40].

Genetically attenuated sporozoites were also evaluated as whole-parasite vaccines, in which the favorite candidates were genetically attenuated, late liver-stage parasites [41]. These parasites are unable to progress beyond the liver stage due to the loss of key genes. This type of vaccine generates a high amount of cross-stage and cross-species protection, and can even offer complete protection when administered by an intradermal or subcutaneous route [42]. Although genetic attenuation has the advantage of avoiding the irradiation step during the production process, it presents other challenges, like the delivery and manufacturing of a cryo-preserved, viable, whole parasite in a vaccine [43].

The *P. falciparum* whole sporozoite vaccine is currently in progress. In 2010, Sanaria Inc. developed a technology to harvest sporozoites of *P. falciparum* from the salivary gland of cultured, parasite-infected mosquitoes [11]. The sporozoites were attenuated using various technologies to make the vaccine. Radiation-attenuated vaccine was called PfSPZ, those attenuated in vivo by anti-malarial drugs were called PfSPZ-CVac, and genetically attenuated vaccine, prepared by gene deletion of essential genes [35], was called PfSPZ-GA1 [36].

Although there are major challenges to develop irradiated sporozoites, this approach offers a high rate of protection (exceeding 90% in trials). However, this efficacy rate was reported with only a few participants [24] and the efficacy in humans was dose-dependent [44–46]. PfSPZ vaccine efficacy showed comparable results with RTS,S vaccine in malaria-endemic settings [29, 47]. Three to five doses of PfSPZ vaccine administered intravenously generated almost 100% protection against homologous, controlled human malaria infection (CHMI), when the NF54 strain was used in naive adults [44, 48, 49]. This regimen also showed a durable but partial protection against heterologous CHMI with 7G8-strain parasites in naive patients [49]. In malaria-endemic areas, a similar dosing in malaria-experienced adults provided more modest immunity against CHMI [50] and naturally occurring malaria [46]. There are several ongoing studies of PfSPZ vaccine in both adults and children.

##### Circumsporozoite protein subunit vaccines

Progress in genetic engineering corresponds with the high efficacy rate reported for whole sporozoite vaccine studies in human. The circumsporozoite protein (CSP) is a protein with a sequence of 412 amino acids; it is a major antigen component on the surface of the malaria sporozoite and is represented early on in the liver phase of infection. Identification of *P. falciparum* CSP led to the cloning and sequencing of the gene coding for the CSP—the first cloned malaria gene [33, 51]. The CSP has continued to be a main focus in protein subunit vaccine development.

***RTS,S vaccine*** The first approved malaria vaccine is RTS,S, a monovalent recombinant protein vaccine that targets a fragment of the CSP. The vaccine contains a truncated CSP of *P. falciparum* that is then fused with the hepatitis B surface (S) antigen, which acts as a carrier for the CS antigen and an immunogenic adjuvant, AS01 [52]. In RTS,S, vaccine, the “R” stands for the central repeat region of the *P. falciparum* CS protein; “T” stands for the T-cell epitope of the CS antigen; the first “S” for “Surface” portion, which when co-expressed on yeast cells display both CS protein and S on their surfaces, while the next “S” stands for the hepatitis B surface antigen. All are assembled in lipoprotein particles (RTS,S) [53].

RTS,S induces a strong IgG antibody response against the conserved central repeat region of the CS protein and potent T-cell (CD4 +) response [22, 54]. Antibody levels reach high concentrations, often of hundreds of micrograms/ml. The levels correlate with the protection from malaria infection or clinical disease in several settings [32, 55]. This vaccine has demonstrated 30–50% protection in field trials in humans in Africa [56, 57]. Based on the pilot results, RTS,S vaccine has been approved by WHO for widespread use in malaria-endemic African countries. It seems that the RTS,S vaccine generates protective immunity and prevents clinical malaria by reducing the merozoites emerging from the hepatic cells. This low number of merozoites reduces the sexual-stage development in the blood cells to a subclinical level, which in turn induces a natural blood-stage immune response and boosts protection [58]. Details of the developmental phases of RTS,S vaccine and its efficacy studies are given in section B.

##### New developments in pre-erythrocytic vaccines

***R21 vaccine*** The R21 vaccine (“next-generation RTS,S-like vaccine”) is an improved version of the RTS,S vaccine developed by the Jenner Institute in Oxford, UK [59]. The R21 and RTS,S vaccines are both virus-like particle-based vaccines based on CSP. R21, however, is formed solely from CSP-HBsAg fusion particles, with a fused CSP-hepatitis B surface antigen. The removal of the unfused S particles is believed to improve the immune response against the CSP, which comprises a higher proportion in R21 than in RTS,S. In addition, R21 was developed to induce a lower immune response against the HBsAg fraction [3]. Both RTS,S and R21 are attached to adjuvants that act as carriers that also boost immunity. However, the adjuvant of the R21 can be more easily manufactured than that of RTS,S, which will hopefully make it cheaper to prepare. The R21 with adjuvant Matrix-M (R21/Matrix-M vaccine) has been developed by Oxford University scientists and has shown an enhanced T-cell response and high protection rate in a Phase II clinical trial on children in a high-malaria-transmission setting [60]. However, questions remain regarding the efficacy of R21 vaccine against CHMI in naive individuals and against naturally occurring malaria in malaria-experienced persons living in endemic areas [3].

***Cell-traversal protein antigen of ookinete and sporozoite (CelTOS) vaccine*** Another antigenic pre-erythrocytic vaccine candidate has been developed using a novel antigen, the cell-traversal protein antigen found in ookinete and sporozoite (CelTOS). This protein antigen was identified as an essential protein for the traversal of *Plasmodium* in mammalian and insect hosts [61]. The evaluation of the CelTOS vaccine candidate in a mouse model revealed a completely sterile immunity against sporozoite challenge [62].

##### Viral-vectored vaccines

The viral-vectored vaccine approach has been used to enhance cellular immunity against the pre-erythrocytic stages [8]. Evaluation of this approach in humans found a strong immune response, mainly from an increased proliferation of CD8-T cells against the viral-vectored-CSP targets. However, the protection rate did not exceed that induced by the RTS,S vaccine [63]. Many vector vaccine generations have been clinically evaluated in trying to promote comparable efficacy [64–66].

The vectors used in this approach included chimpanzee adenoviruses [64], boosted by the modified vaccinia virus Ankara [67]. This boosted approach, used here for the first time in vaccines, resulted in an improved T-cell immune response compared to using only one viral vector [67–69]. Other vectors used included the adenoviruses Ad35 and Ad 26 [70], which, like other chimpanzee viruses, resist the harmful effects of a naturally acquired immune response to human adenoviruses.

The viral-vectored pre-erythrocytic vaccines have encompassed various protein antigens including CSP, and thrombospondin-related adhesion protein (TRAP). Blood-stage antigens such as merozoite surface protein-1 (MSP1) and apical membrane antigen-1 (AMA1) have also been tried. Another approach used plasmid DNA as priming vector, and a human adenovirus, Ad5, to boost the immune response [65]. Several antigenic inserts from both pre-erythrocytic stage and blood stages showed encouraging efficacy.

##### Challenges facing the development of circumsporozoite protein vaccines

The targeted CSP antigens of the vaccines, as in many *Plasmodium* antigens, have shown antigenic variation, including the targeted antigen of the RTS,S C-terminal region. In a Phase III trial on RTS,S, better efficacy was seen against parasites that had a matched sequence with the C-terminal region of the vaccine sporozoites [30]. This means that the unmatched parasite variants may escape the vaccine’s action and may continue to spread in the community. Another challenge for the RTS,S vaccine is its structure, which does not include an N-terminal region of the CSP that is crucial for the attachment to and invasion of the sporozoites into the liver cells [71]. The N-terminal region has shown induced natural immunity associated with malaria protection in African children [72]. Improved CSP-vaccines are being developed to prime vaccine-immune response by selecting various antigenic epitopes that show protective antibodies [17]. The fact that whole sporozoite vaccines induce better protection than subunit vaccines [24, 73] suggests that antigen-combination strategies are necessary. Further research into other potential malaria vaccine antigens and strategies for their delivery is therefore essential [52].

#### Erythrocytic vaccines (blood-stage vaccines)

These vaccines act when the merozoites are released from the liver (after completion of the pre-erythrocytic stage) and enter the blood to infect erythrocytes. Hence, these vaccines are also referred to as blood-stage vaccines. Their goal is to block the invasion of red blood cells by the merozoites, prevent the parasite’s asexual reproduction and to elicit anti-invasion and anti-disease responses [74]. These blood-stage vaccines induce antibodies to the surface antigens of the merozoites and against variant antigens on the red blood cell membranes [75–77]. Unlike the promising progress in the pre-erythrocytic vaccines, progress in erythrocytic vaccines has been slow [78]. Development of the blood-stage vaccines faces many challenges, including the very short time that the merozoites are freely available outside the erythrocytes for easy attack by the induced antibodies, the large number of merozoites that need to be targeted compared with the low number of sporozoites in the pre-erythrocytic phase, the antigenic diversity, and the many invasion pathways [17]. How to address genetic polymorphism is an important issue to explore for this group of vaccines. It has been suggested that efforts should concentrate on antigens or constructs inducing cross-reactive immune responses, which would cover genetic diversity.

Several blood-stage antigens have already been tried: erythrocyte-binding antigen-175 (EBA-175) [79], apical membrane antigen-1 (AMA-1) [80], glutamate-rich protein (GLURP) [81, 82], serine repeat antigen 5 (SERA5) [83, 84] and merozoite surface protein (MSP-1) [85], MSP-2 [86], and MSP-3 [87, 88]. All these antigens are highly expressed on the surface of the merozoites, but have not shown a significant impact on clinical malaria. After these disappointments, other antigens with strong immunogenicity and great potential as blood-stage vaccine candidates were suggested. For example, the merozoite antigen, *P. falciparum* reticulocyte-binding protein homologue 5 (PfRH5) has been shown to generate neutralizing antibodies that target its common genetic variants [89, 90]. However, PfRH5 has exhibited limited polymorphism and pre-clinical studies showed that the antigen is the first, very conserved blood-stage antigen that generates broadly inhibiting antibodies [90]. Notably, natural infections induce modest or no antibody against PfRH5 [90–92]. In addition, rhoptry-associated leucine zipper-like protein-1 (RALP-1), which plays an important role during merozoite invasion into erythrocytes, has been suggested as a target [93]. Another new blood-stage vaccine, a combination of AMA-1 with the rhoptry-neck protein RON2 (AMA1-RON2) has attracted interest because its binding at the merozoite–erythrocyte junction induces cell invasion. However, AMA1-RON2 showed low efficacy in previous studies. This combined antigen can induce improved immunogenicity of non-combined AMA-1 antigen with more effective anti-invasion inhibitory antibodies [94].

Other new blood-stage vaccine antigens include those parasite antigens that are expressed on the infected red blood cells; these stay available for hours to be targeted by the induced antibodies. Of these, the PfEMP1 is an immunodominant virulence antigen that facilitates sequestering of the *P. falciparum* parasites and is targeted by naturally acquired immunity [95]. No further progress has been made with the PfEMP1 vaccine because the antigen is large and has high genetic polymorphism, with a complicated structure of cysteine-rich content. No evaluations have assessed PfEMP1-vaccine efficacy.

Another erythrocyte surface protein, called PfGARP, has been described as a target for protective antibodies [96] and *P. falciparum* Schizont Egress Antigen-1 (PfSEA-1), which emerges from infected blood cells, has also been identified [97]. After repeated disappointments with various blood-stage vaccine candidates, scientists have tried other erythrocytic-stage antigens which are chemically attenuated by culturing with a DNA-binding agent, tafuramycin-A. These attenuated erythrocytic-phase parasites (CAP) induce homologous as well as heterologous immunity in mice, and their protection depended on CD4 + T cells [98–100].

#### Transmission blocking vaccines (TBVs) (mosquito stage vaccines)

TBV vaccines aim to induce antibodies against functionally important proteins that are expressed on developmental stages of the parasite in the mosquito [101]. They target antigens on parasite gametes, zygotes and ookinetes [52]. The TBVs block the infection transmission from human to mosquito and so prevent malaria spreading [102]. These vaccines generate antibodies that prevent the *Plasmodium* sexual reproduction in the mosquito by blocking either the fertilization of the gametes, the transition of ookinete-to-oocyst, the development of the zygote into sporozoites [103, 104], or the sporozoites' invasion of the salivary gland [105].

The main transmission blocking vaccine candidates that are currently being developed include Pfs-25, Pfs-48/45, and Pfs-230 [17, 106]. Both Pfs-48/45 and Pfs-230 are gametocyte-expressed antigens that are present in human and mosquito vectors and continue forming as a protein complex on the *P. falciparum* gamete surface [107]. The antibodies formed against the gametocyte and its Pfs-230 and Pfs-48/45 antigens during the naturally acquired immune response have induced transmission blocking activity [108].

The major limitation of TBVs is that they do not protect the recipient from contracting malaria as they do not impede the infection route. They might be helpful in reducing disease transmission in the long run, after mass immunization has been achieved. So they could benefit the whole community and hence the terms ‘community vaccine’ and ‘altruistic vaccination’ are becoming popular [8]. However, this approach is unattractive for individuals or for Western travelers, who are the major driver of vaccine development efforts [10].

Another important limitation of TBVs is their low efficacy, because human immune mechanisms are not naturally exposed to TBV candidate antigens, and thus the boost to immunity is limited [109]. Some have proposed that malaria might adapt to a new vector, or to an alteration of certain protein compounds required for interaction with the vector [10]. In addition, because TBVs should target all individuals (including children and infants) who can transmit the disease to accomplish herd immunity, this type of mass vaccination would pose a major logistical challenge [8]. Furthermore, TBVs must have an exceptional safety profile, since they do not confer a direct benefit to the individual [17]. Hence, some have recommended their application be combined with efficacious pre-erythrocyte vaccines to prevent both infection in humans and transmission to mosquitoes, and these could also be combined with blood-stage vaccines that would add a synergistic effect by reducing onward transmission [101, 110]. Nevertheless, TBVs could still be important tools in malaria elimination and eradication programs, for preventing transmission [111].

##### *Plasmodium vivax* vaccines

Although most research and funding efforts have so far been dedicated to developing *P. falciparum* vaccines, *P. vivax* vaccine also deserves attention. *P. vivax* forms an important public health problem, with a high burden and high rates of morbidity and mortality in many settings [112]. In addition, *P. vivax* has been shown to induce sterile heterologous immunity in human studies [113, 114]. Some promising attempts have been made to develop a *P. vivax* vaccine, including a pre-erythrocytic vaccine of circumsporozoite protein (Pv-CSP), a blood-stage vaccine of merozoite Duffy Binding Protein (Pv-DBP), and transmission blocking vaccines (Pv-s25) [115–117]. These candidates have progressed to pre-clinical and clinical trials with promising results. Viral-vector vaccines [116] as well as recombinant antigen [117] approaches have also been used with Pv-DBP. Good transmission blocking was reported with Pv-s25 with a well-tolerated and modest antibody response in mosquito studies [115]*.*

**Fig. 2:**
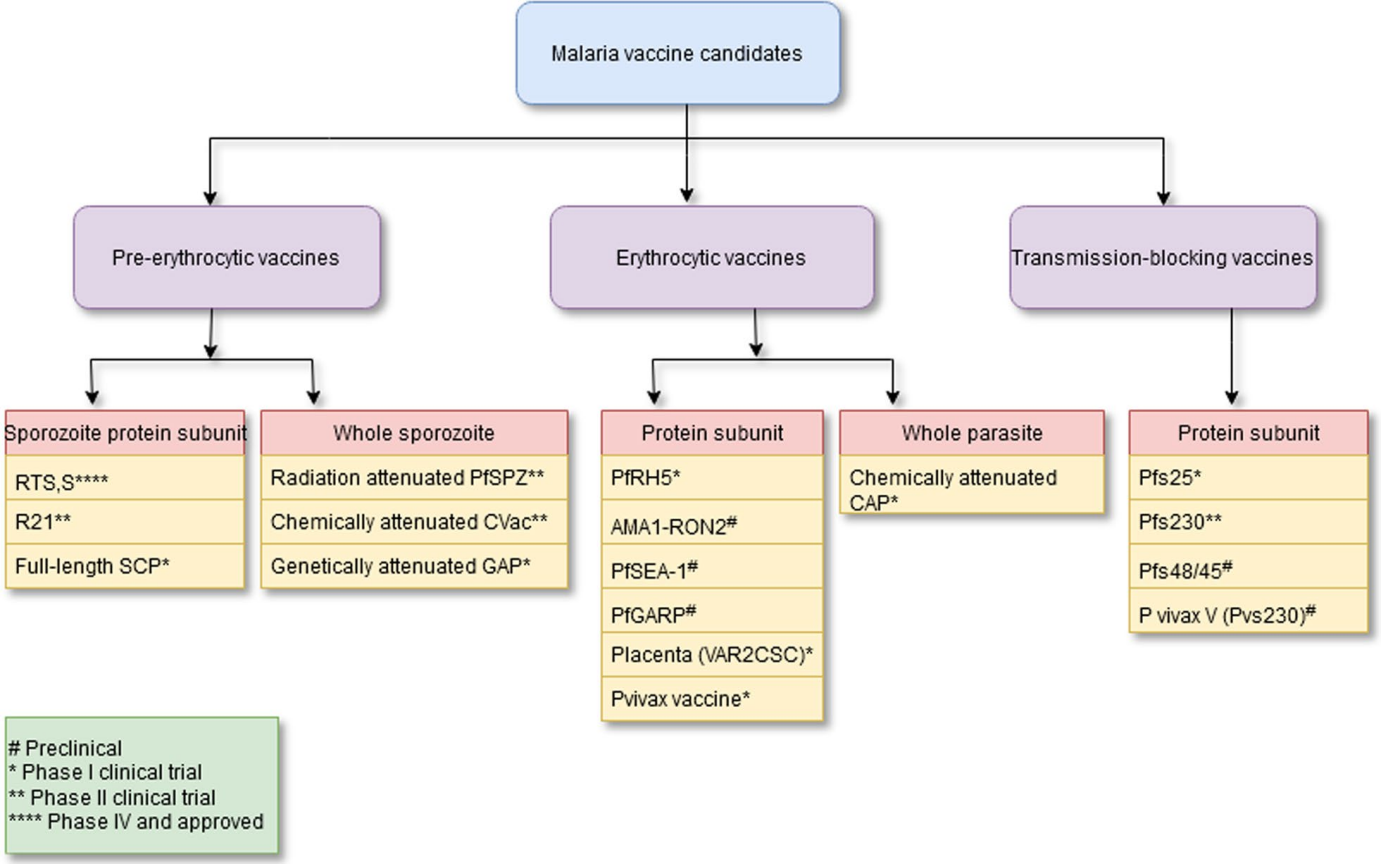
Summary of malaria vaccine candidates with their type and developmental phase. See reference [17]

**Fig. 3:**
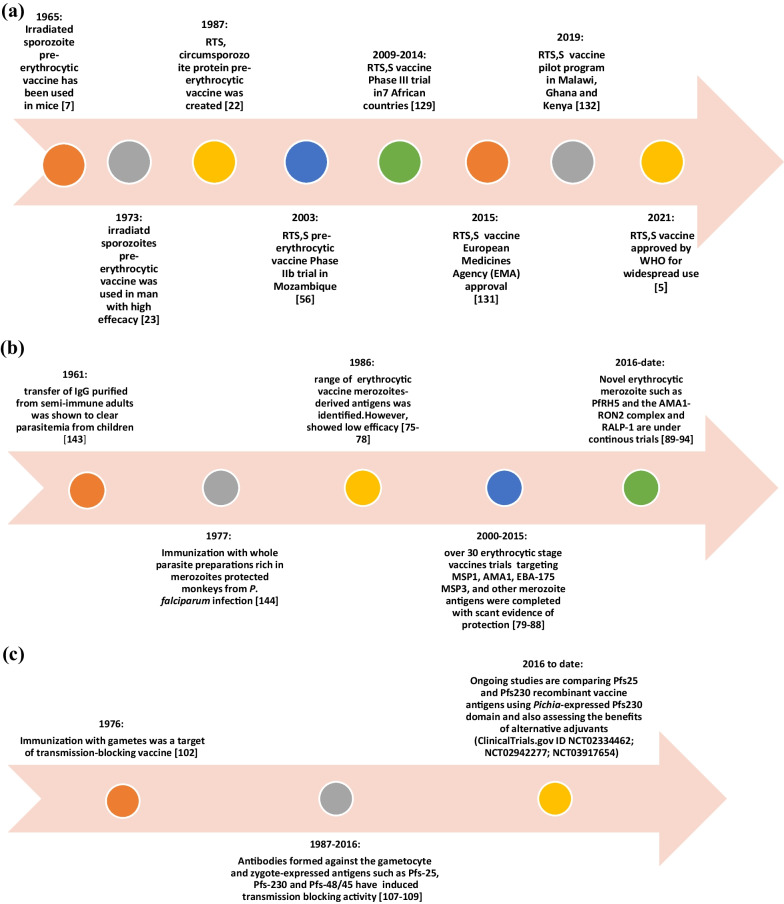
**a** A chronological diagram showing the main milestones in the development of the pre-erythrocytic malaria vaccines. **b** A chronological diagram showing the main milestones in the development of the erythrocytic malaria vaccines. **c** A chronological diagram showing the main milestones in the development of the transmission-blocking malaria vaccines

Figure 2 shows a summary of the malaria vaccine candidates with their type and developmental phases [17]. A timeline of the major turning points in the creation of pre-erythrocytic malaria vaccinations is shown in Fig. 3a. The major turning points in the creation of erythrocytic malaria vaccines are shown in Fig. 3b. Figure 3c depicts a timeline of the significant turning points in the creation of transmission-blocking vaccinations.

### The first approved malaria vaccine: history of RTS,S vaccine development

The first pre-erythrocytic RTS vaccine was created in 1987 and, by 2019, it was tested in a pilot program in seven African countries with endemic malaria. In 2021, RTS vaccine was the first malaria vaccine approved for widespread use. Figure 3a shows the main steps in the development of RTS,S vaccine from creation to approval.

#### RTS,S—creation and early evaluation

The RTS,S vaccine was created in 1987, as a result of a collaboration that began in 1984 between the multinational pharmaceutical company GSK and the Walter Reed Army Institute of Research (WRAIR, Maryland, U.S.A.) [22]. At that time both groups were trying to create a vaccine, based on studies that proved good efficacy of attenuated-sporozoites in protecting against malaria [23]. Other pre-clinical studies revealed that CSP inoculation could induce antibodies that protected against active *P. falciparum* infection [118]. This vaccine was effective only against *P. falciparum* but not against *P. vivax* or any other types of malaria [33]. The first attempt to develop RTS vaccine was to identify the CSP antigen as a target of the immune response generated by radiation-attenuated sporozoites. The U.S. National Institutes of Health (NIH) and WRAIR [51, 119] then cloned and sequenced this antigen. They found it difficult to produce a whole-length CSP antigen, so they replaced it by using GSK’s *Escherichia coli* elaboration system and produced a central-repeat region subunit antigen [120].

The initial structure of the vaccine did not yield good efficacy. It was formed from a CSP-tandem-repeat region, chiefly the NANP 4-amnio acid sequence [26]. Expressing the central repeat (R), a single polypeptide chain corresponding to a highly conserved, tandem repeat tetrapeptide NANP amino acid sequence, fused to the C-terminal region known to contain T cell epitopes (T). To add a carrier to the central repeat region, the RT particle was fused to the hepatitis-B surface antigen (S), yielding a yeast-expressed protein RTS [54]. However, to generate immunogenic particles, this protein needed to be co-expressed with large amounts of the unfused S protein. Another unfused hepatitis-B surface antigen portion was added—a second (S)—that spontaneously fuses to the RTS component, hence the name became RTS,S. Then they tried to add many adjuvants to the vaccine. In 1996, a key study assessed several adjuvants and found that the highest efficacy was obtained with the RTS,S vaccine which had an adjuvant that contained monophosphoryl lipid-A, which is an immune stimulant agonist of toll-like receptor 4, and a Quill A derivative [54]. When using this adjuvant (called AS02) and a related adjuvant (AS01), the RTS,S vaccine produced a protective efficacy of 30–50% in healthy participants challenged by sporozoites in a series of studies. The AS01 adjuvant showed higher protection than AS02, with higher numbers of antibodies against CSP and a higher CD4 + T-cell response in naive participants in a CHMI study [121]. These findings were confirmed by a study in Kenya [47]. This RTS,S/AS01 formulation of the vaccine was subsequently tested as part of Phase II and Phase III trials and in the implementation program [122–126]. Their results led to the RTS,S vaccine being approved for widespread use to protect against *P. falciparum* malaria in African countries.

#### RTS,S Phase II clinical trials

In 2003 to 2004, and encouraged by earlier results that demonstrated the strong immunogenicity of RTS,S vaccine, a Phase IIb, double-blind randomized controlled clinical trial was implemented with more than 2000 children aged 1 to 4 years in Mozambique [56]. In this trial, three vaccine doses reduced the incidence of malaria by 37% compared to the control group in the 6 months following the third dose. The efficacy of the vaccine was estimated as 1 minus the ratio of the incidence rates in each group. For all clinical events, efficacy was 27%, while for severe cases it was 58%. The 12-month follow-up raised efficacy to 29% for all malaria cases, whereas it dropped to 39% for severe cases. The 18-month follow-up had an efficacy of 35% for all malaria events and 49% for severe malaria. While the children’s young age in this trial has shown no association with RTS,S efficacy, later Phase IIb clinical trials in African infants has shown an efficacy of 65% in the 6 months following RTS,S vaccination [122, 127, 128].

#### RTS,S Phase III clinical trials

The encouraging results of the early trials on RTS,S/AS01 vaccine led to a Phase III randomized trial from 2009–2014 (conducted by a collaboration between a private foundation, a vaccine manufacturer, and a public health agency). The Phase III trial enrolled 15,459 children at 11 locations in seven African countries (Burkina Faso, Ghana, Malawi, Gabon, Kenya, Mozambique and Tanzania) [129]. The vaccine was delivered as three doses of 0.5 mL and administered intramuscularly at monthly intervals, followed by a fourth dose (booster) 18 months after the third dose. The primary end point was clinical malaria events, which were reduced by almost 26% of the pre-trial rate in infants and by almost 36% in young children after four doses. Vaccinated young children, rather than infants, also showed protection against severe malaria. However, the efficacy of the RTS,S vaccine declined with time and clinical malaria events dropped to 68% of the pre-trial rate in the first six months [130].. RTS,S is about 56% effective over one year and 36% effective over four years. The prevented malaria events were 1774 in 1000 children who were on the 4-dose regime and 1363 in 1000 children who were on the 3-dose regime [129, 130]. Most of the prevented events were reported in high transmission settings. However, the vaccine’s efficacy was estimated to be higher in settings of low incidence, but the difference was not statistically significant.

#### The 2015 milestone: approval by the European Medicines Agency

In 2015, the Committee for Medicinal Products for Human Use (CHMP) of the European Medicines Agency (EMA) documented that the RTS,S/AS01 vaccine had an acceptable safety profile that was to be continually monitored [131, 132]. The CHMP gave a supporting scientific decision for use of RTS,S outside the European Union “in areas where malaria is regularly found, for the active immunization of children of six weeks up to seventeen months old against malaria caused by the *P. falciparum* parasite, and against hepatitis B”[131].

In late 2015, two main WHO groups, the Strategic Advisory Group of Experts (SAGE) and the Malaria Policy Advisory Group (MPAG), reviewed the findings of the Phase III clinical trial on RTS,S. In January 2016, and based on the recommendations of both advisory groups, the WHO approved a pilot implementation program on RTS,S vaccine in three moderate and high-transmission African countries using the four-dose protocol. The pilot program was started in 2019.

#### RTS,S 2019 pilot program

In April 2019, the WHO launched the RTS,S pilot program in three African countries (Malawi, Ghana, and Kenya) to assess vaccine effect on childhood mortality, its safety during routine use in the national immunization programs, and the feasibility of delivering four doses to children [132]. The three-dose intramuscular vaccination schedule for infants was performed at 6, 10, and 14 weeks of age. For older children, the three monthly doses were started at 5–17 months old. The fourth booster dose was given 18 months after the third dose in all age groups [132]. The vaccine was administered through the routine national immunization program, coordinated by the Ministry of Health in each country.

Up to the end of September 2021, and despite the COVID-19 pandemic, over 800,000 children were included in the pilot program [133]. Together with the results of earlier clinical trials, the key findings from these three countries have informed the WHO’s decision on RTS,S vaccine. In October 2021, the WHO recommended the widespread use of the vaccine for children in moderate–high transmission settings in Africa and other places [133].

#### Summary of the key findings of the pilot program

The pilot program has revealed a high uptake of the RTS,S vaccine and re-confirmed its positive safety profile. RTS,S has significantly reduced severe malaria, life-threatening incidence, and children’s hospitalization due to malaria. The pilot has generated evidence and experience on the feasibility, impact and safety of the vaccine in routine, real-life situations. The pilot has also yielded the following findings [133]:

- RTS delivery was feasible despite the COVID-19 pandemic and equity in the vaccine coverage was achieved everywhere as part of routine child immunization programs.
- RTS has been reaching (very nearly) all vulnerable children. The introduction of RTS,S has increased the percentage of children reached by malaria prevention methods to over 90% (insecticide-treated nets or RTS,S vaccine). In the three countries, more than two-thirds of those who were not using bed-nets benefited from the vaccine.
- RTS showed a good safety profile: up to October 2021, the number of administered doses exceeded 2.3 million in the three countries with advantageous safety outcomes.
- RTS introduction has not negatively affected bed-net use, the child vaccination programs, or people seeking healthcare for other febrile diseases.
- RTS has had a major effect on real-life child vaccination settings: the vaccine reduced fatal and severe malaria events by 30%, even in settings that widely used bed-nets for prevention and in the presence of good malaria healthcare.
- RTS is highly cost-effective**:** modeling studies have shown that RTS is cost-effective in endemic settings.

The pilots are planned to continue through 2023 in these three countries, with the aims of evaluating the outcome of the fourth dose and to assess its effect on child mortality in the longer term [133].

### RTS,S supporters

Thirty years of collaboration in research and development between GSK, PATH global health organization, and African research partners have led to the RTS malaria vaccine. The generous funding from the Bill & Melinda Gates Foundation in 2001 through 2015 catalyzed the later stage of the vaccine development [133]. The pilot program launched in 2019 was financially supported by a significant collaboration between Vaccine Alliance, Gavi, Unitaid, and the Global Fund to Fight AIDS, Tuberculosis and Malaria [133]. The pilot program was also supported and coordinated by many national and international partners, including the WHO, UNICEF, PATH and GSK (GSK donated ten million RTS,S vaccine doses). National consortiums of evaluation partners collected the data for each pilot program to inform the WHO [133].

### Future prospects for successful malaria vaccines

There are new malaria vaccines on the horizon, features of good malaria vaccine are outlined, and the next steps required for the approved and developing vaccines are discussed in the sections below.

#### New malaria vaccines on the horizon

There are two main *P. falciparum* vaccines at an advanced stage of development, R21 and PfSPZ. They are being continually tested in clinical trials in naive and experienced malaria participants for both safety and efficacy. The two vaccines are included in the WHO-Rainbow Tables, along with other candidates [134] and have recently been reviewed [135, 136]. In addition to R21 and PfSPZ vaccines, BioNtech efforts to develop a vaccine based on mRNA technology are ongoing, inspired by their success in COVID-19. This approach may be an answer to the challenges facing malaria vaccine development, which include the evasion of immune mechanisms by the malaria parasite [60]. It is hoped that an mRNA malaria vaccine will have high efficacy be easily manufactured, and safe for all individuals.

#### Features of ideal malaria vaccine

Many experts have suggested that a highly effective vaccine is likely to include antigens from multiple stages of the parasite’s life cycle. It is hoped that the multi-component vaccine suggested will induce an effective and sustainable protective response [137]. The multi-component vaccine should generate protection against sporozoites, sexual and asexual stages, and also against infected liver cells. This vaccine should also elicit different types of immune reaction, i.e. humoral and cell-mediated responses. In addition, to conquer the antigenic and genetic variations, the vaccine should include several epitopes that are represented by various molecules of the major histocompatibility complex (MHC) [137]. However, there are still some challenges that may impede development of the multi-component vaccine, including increased cost of manufacturing, unless it can be given by a single delivery approach like the pox-viral vector [138, 139].

An example of a combination vaccine is to combine a protein/adjuvant vaccine, specifically RTS,S, that induces antibodies to clear sporozoites before they can enter the liver, and vectored vaccines that clear infected liver cells through activation of T cells. When administered as a simple mixture, the two vaccines have shown to provide 90% sterile efficacy [140]. The RTS,S vaccine can reduce over 95% of the sporozoites before they enter the liver cells, while the vector vaccine can reduce the number by more than 90%. The synergistic effect of both vaccines, based on what has already been reported in clinical trials for each individual vaccine, would speed up the development of the highly effective vaccine [8]. Besides being highly effective, the ideal malaria vaccine should also be safe, stable under various conditions such as temperature, light and transportation, easy to administer, and must provide long-term immunity. Such vaccines should also be cost-effective and affordable in poor malaria-endemic areas [141].

#### Next steps on the road toward successful malaria vaccines

Now the WHO has finally approved the wider utilization of the RTS,S vaccine, the question remains how well the vaccine will work over a wider area and how we can best utilize it to benefit the malaria-endemic communities. The potential impacts of the vaccine on health status, childhood mortality, poverty, and social justice for people living in endemic areas are important issues that need to be monitored. Therefore, evaluations are required to measure these long-term impacts of the vaccine [133].

Further steps may also include decisions on funding opportunities that will be very important in defining how broadly the vaccine can be used in the most needy communities and in determining national decisions on adopting the vaccine in endemic countries. An operational guide is also needed to lead countries through what is required to integrate the malaria vaccine into the national immunization program and its use alongside other preventive tools like bed-nets [60].

The RTS vaccine has enabled us to meet the first target of the Malaria Vaccine Technology Roadmap that was published in 2006 [142]; it is a first-generation vaccine with at least 50% efficacy lasting for one year. Further, we look forward to meeting the second target of the roadmap, which is to have a second-generation vaccine with at least 80% efficacy lasting four years by 2025. There is ongoing work to develop extra types of malaria vaccines and a variety of vaccine candidates are showing promise for the 2025 target [8]. The recently developed pre-erythrocytic vaccine candidates like PfSPZ, R21 and full-length circumsporozoite protein immunogens are being improved in efficacy [17]. At the same time, the transmission blocking vaccines have progressed to advanced-phase trials. Combining both transmission blocking vaccines with pre-erythrocytic vaccines like RTS with other tools of malaria control would certainly benefit the malaria eradication programs [17]. Future advances for the RTS,S vaccine may include improved protection through a schedule of fractionated delayed doses and alternative adjuvants.

Unfortunately, blood-stage vaccines that target merozoite invasion proteins have so far delivered disappointing efficacy. Novel targets of blood-stage vaccines, like infected erythrocytes’ surface proteins, egress antigens that emerge from schizonts and attenuated, intact infected red blood cells, continue to be developed [17]. In addition, the substantial progress made with *P. falciparum* vaccine justifies increased efforts and investment in *P. vivax* vaccines to pursue similar goals and to achieve the ultimate aim of malaria eradication [17].

**Fig. 4:**
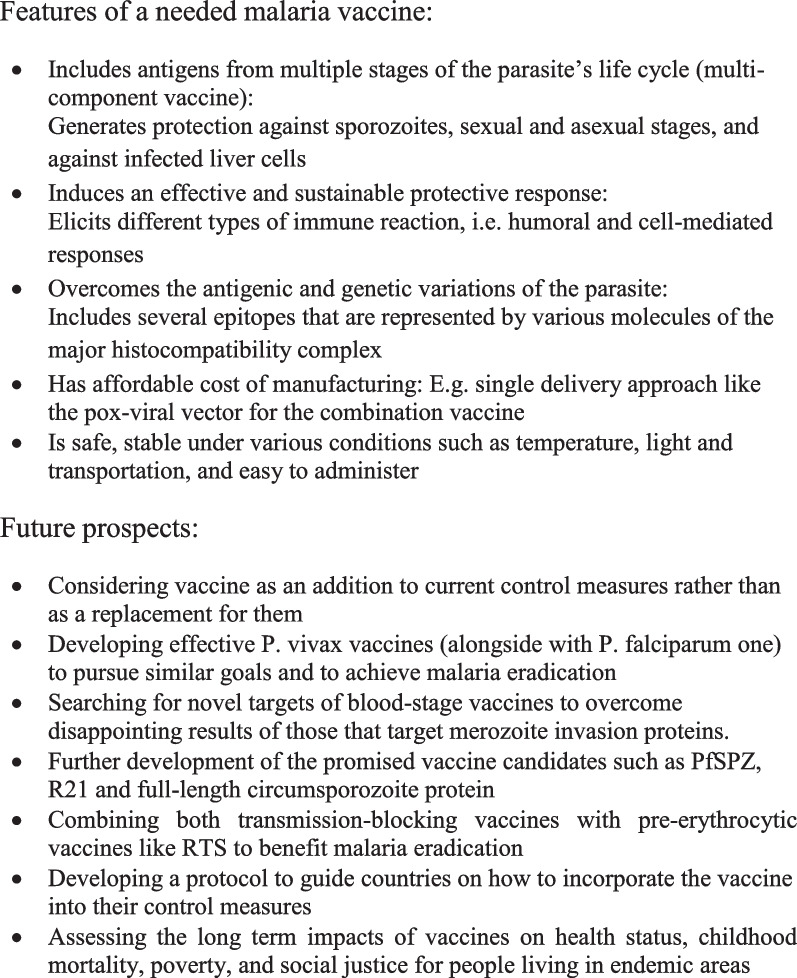
Features of a needed malaria vaccine and prospects for successful malaria vaccines

Finally, although successful vaccines such as RTS,S/AS01 have proven to prevent clinical malaria in immunized individuals, they might not be sufficient as a stand-alone measure for global malaria eradication. These vaccines should be taken as an addition to current control measures rather than as a replacement for them. A protocol on how to incorporate the vaccine into other control measures, to eradicate malaria successfully is being developed by the WHO [60]. Figure 4 summarizes the future prospects for successful malaria vaccines, itemizing the conditions for which malaria vaccines are needed.

## Conclusion

After almost 60 years of struggling to achieve the dream of having an efficacious vaccine as a tool to fight malaria and to conquer its enormous burden, the long awaited moment finally arrived in 2021. The complicated life cycle of *Plasmodium*, its genetic diversity, and the absence of sterile immunity in malaria has long presented a challenge to malaria vaccine development. Modern malaria vaccine development stemmed from studies in the 1960s that immunized mice with irradiated sporozoites. There was continual progress on malaria vaccine candidates. The first-ever malaria vaccine (and also the first parasite vaccine), RTS,S AS01 was approved for widespread use on 6 October 2021. The WHO recommended the broader use of the vaccine among children at risk in African countries and in other areas where *P. falciparum* has high or moderate transmission. This decision was justified by the favorable efficacy and safety results of the RTS vaccine in Phase II and III clinical trials and in a pilot program conducted in three African countries. The studies showed that RTS was about 56% effective over one year and 36% effective over four years, with an acceptable safety profile. The questions now remain how well RTS,S vaccine will work with widespread use, what are its long-term impacts on child health and on targeted communities, and how can we benefit the most from its use in the worst affected and endemic communities.

Nonetheless, we still need the newer vaccine candidates—including pre-erythrocytic, erythrocytic and transmission-blocking vaccines—to be developed. A multi-component vaccine that increases the probability of a sustainable and effective host response may prove very promising. More efforts and investment in *P. vivax* vaccines should also be encouraged, in order to attain global malaria eradication.

Finally, although RTS,S vaccine has been approved for wider use in endemic African countries and elsewhere, it might not be sufficient as a stand-alone measure for effective malaria control. In order to achieve malaria elimination, it is wiser to consider the vaccine as an addition to current measures rather than as a replacement for them. A protocol to guide countries on how to incorporate the vaccine into their control measures is being developed.

## Data Availability

Not applicable.

## Abbreviations

WHO: World Health Organization
GSK: GlaxoSmithKline
P: Plasmodium
PATH: Global health organization (formerly Program for Appropriate Technology in Health)
PEVs: Pre-erythrocytic vaccines
CSP: Circumsporozoite protein
WSV: Whole sporozoite vaccine
CHMI: Controlled human malaria infection
TBVs: Transmission blocking vaccines
DBP: Duffy Binding Protein
WRAIR: Walter Reed Army Institute of Research
NIH: National Institutes of Health
CHMP: Committee for Medicinal Products for Human Use
EMA: European Medicines Agency
SAGE: Strategic Advisory Group of Experts
MPAG: Malaria Policy Advisory Group
COVID-19: Coronavirus disease of 2019
UNICEF: United Nations Children's Fund
MHC: Major histocompatibility complex
